# Case report: response to immunotherapy and association with the fh gene in hereditary leiomyomatosis and renal cell cancer-associated renal cell cancer

**DOI:** 10.1186/s12920-024-01957-w

**Published:** 2024-08-19

**Authors:** Fangfang Gao, Dejian Gu, He Zhang, Chao Shi, Feng Du, Bo Zheng, Huijuan Wu, Yanqiu Zhao

**Affiliations:** 1 Department of Internal Medicine, The Affiliated Cancer Hospital of Zhengzhou University, Henan Cancer Hospital, No. 127 Dongming Road, Zhengzhou, Henan 450008 China; 2 Geneplus-Beijing Co., Ltd, Beijing, China; 3 Department of Pathology, The Affiliated Cancer Hospital of Zhengzhou University, Henan Cancer Hospital, Zhengzhou, Henan China; 4 Department of Molecular Pathology, The Affiliated Cancer Hospital of Zhengzhou University, Henan Cancer Hospital, Zhengzhou, Henan China; 5 Department of Radiology, The Affiliated Cancer Hospital of Zhengzhou University, Henan Cancer Hospital, Zhengzhou, Henan China

**Keywords:** HLRCC-RCC, FH gene, Immunotherapy, PD-L1

## Abstract

Hereditary leiomyomatosis and renal cell cancer (HLRCC) is a rare autosomal dominant syndrome caused by a germline mutation in the fumarate hydratase (FH) gene that manifests with cutaneous leiomyomas, uterine fibroids, and renal cell cancer (RCC). Patients with HLRCC-associated RCC (HLRCC-RCC) have aggressive clinical courses, but there is no standardized therapy for advanced HLRCC-RCC. In this study, we described a case of aggressive HLRCC in a 33-year-old female who exhibited a novel heterozygous germline insertion mutation in exon 8 of the FH gene (c.1126 C > T; p.Q376*). The patient underwent laparoscopic resection of the right kidney, but metastases appeared within 3 months after surgery. Histological staining of the resected tumor revealed high expression levels of programmed cell death-ligand 1 (PD-L1). Therefore, the patient was treated with immunotherapy. The patient achieved a partial response to immunotherapy, and the treatment of metastatic lesions has continued to improve. A thorough literature review pinpointed 76 historical cases of HLRCC-RCC that had undergone immunotherapy. From this pool, 46 patients were selected for this study to scrutinize the association between mutations in the FH gene and the effectiveness of immunotherapy. Our results indicate that immunotherapy could significantly improve the overall survival (OS) of patients with HLRCC-RCC. However, no influence of different mutations in the FH germline gene on the therapeutic efficacy of immunotherapy was observed. Therefore, our study suggested that immunotherapy was an effective therapeutic option for patients with HLRCC regardless of the type of FH germline mutation.

**Supplementary Information:**

The online version contains supplementary material available at 10.1186/s12920-024-01957-w.

## Introduction

Hereditary leiomyomatosis and renal cell cancer (HLRCC) is an autosomal dominant condition (although incomplete penetrance is also possible) characterized by the development of cutaneous leiomyomas, multiple uterine leiomyomas, and an aggressive form of type 2 papillary renal cell cancer (RCC) [1–3]. HLRCC is caused by germline mutations of the fumarate hydratase (FH) gene, which is detected in 90% of HLRCC families [4]. Approximately 15% of individuals with FH mutations develop HLRCC-associated RCC (HLRCC-RCC), which was defined as a distinct entity in the 2016 World Health Organization classification [5].

FH acts as a tumor suppressor gene that encodes fumarate hydratase, which is an enzyme that catalyzes the conversion of fumarate to malate in the Krebs cycle [6]. Mutations in the FH gene, including missense, frameshift, and complete or partial deletions, can cause a decrease in the expression of fumarate hydratase, causing intracellular fumarate accumulation and leading to cancer [7]. The accumulation of fumarate due to FH gene mutations had led to its role as an electrophile, which spontaneously reacted with the thiol groups of cysteine, a process known as succination. These reactions resulted in alterations to the activity of numerous proteins, leading to the dysregulation of associated pathways and, ultimately, the formation of HLRCC tumors [8]. Accurate identification of pathogenic germline variants in the FH gene is helpful for disease management and monitoring of patients and their family members [9].

Patients with HLRCC-RCC usually have a poor clinical course. There is no standard therapy or consensus for treating advanced HLRCC-RCC. Several studies have reported that immunotherapy might improve the prognosis of HLRCC patients, but its efficacy has fluctuated across studies [10–12]. In a recent Phase II study of the combination therapy of Cabozantinib plus Nivolumab for patients with RCC, all five patients with HLRCC responded to the treatment [12]. Additionally, multicenter study analyses have indicated that patients with HLRCC who receive immunotherapy-based treatments have better overall survival and progression-free survival in the first line of treatment [10]. Furthermore, in two cases of HLRCC with Programmed Cell Death-Ligand 1 (PD-L1) positivity, the efficacy of immunotherapy was also reported [13, 14].Here, we describe a case of aggressive HLRCC in a 33-year-old female who exhibited a novel heterozygous germline insertion mutation in exon 8 of the FH gene (c.1126 C > T; p.Q376*) and had positivity of PD-L1. She has benefited from immunotherapy treatment with a partial response and her disease has remained stable for over 20 months. Then, we reviewed HLRCC-RCC patients who received immunotherapy and analyzed the association between mutations of the FH gene and the benefit of immunotherapy. Our study suggested that immunotherapy is an effective therapeutic option for patients with HLRCC-rcc regardless of the type of FH germline mutation.

## Case presentation

**Fig. 1:**
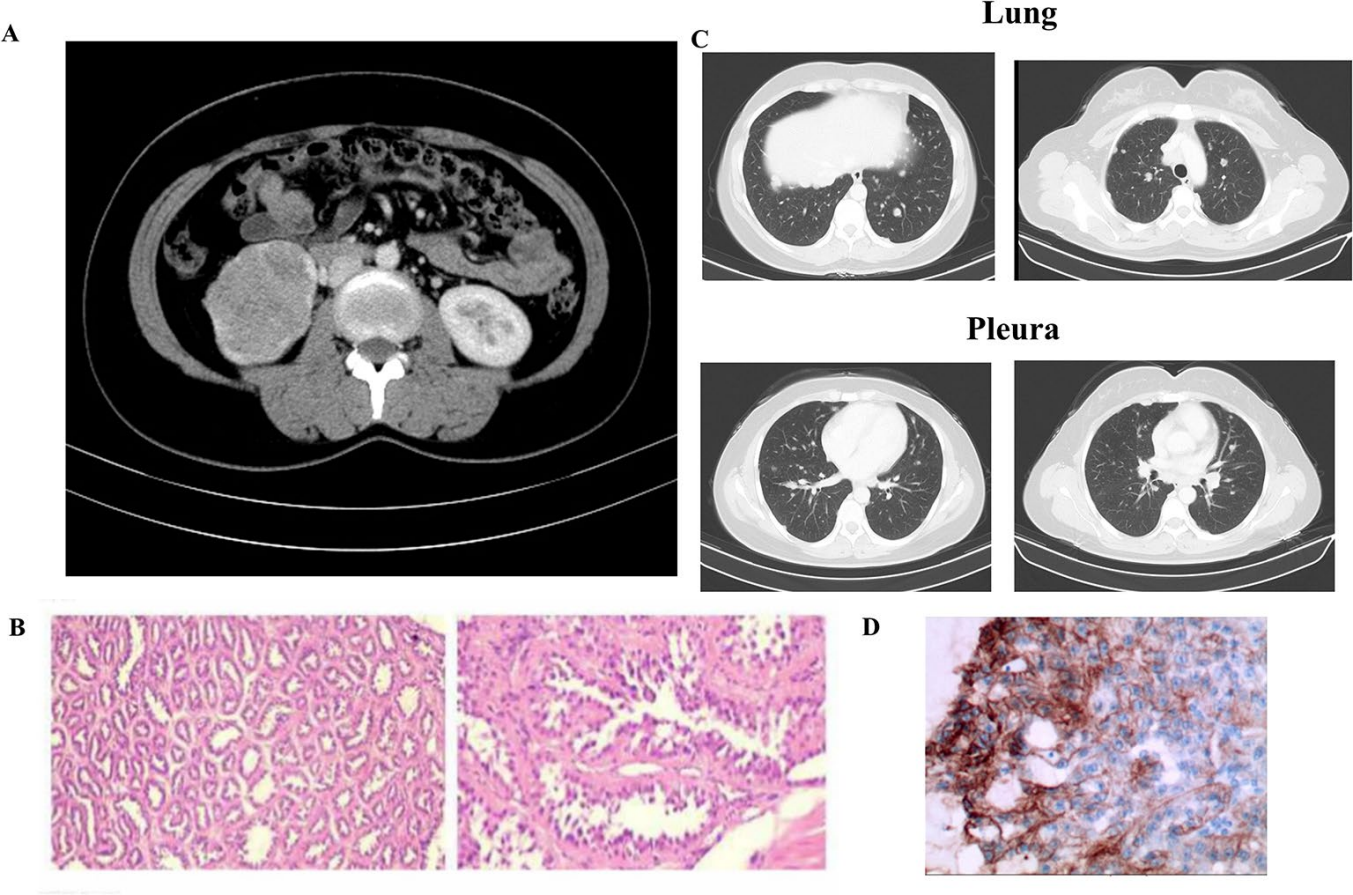
Diagnosis of hereditary leiomyomatosis and renal cell cancer-associated renal cell carcinoma (HLRCC-RCC). **A**. CT revealed a large mass in the right kidney. **B**. Hematoxylin–eosin staining showed renal cell carcinoma with papillary and tubular structures. **C**. CT revealed multiple nodes in both lungs and the pleura. **D**. Approximately 20% of the tumor cells exhibited programmed cell death-ligand 1 (PD-L1) expression

**Fig. 2:**
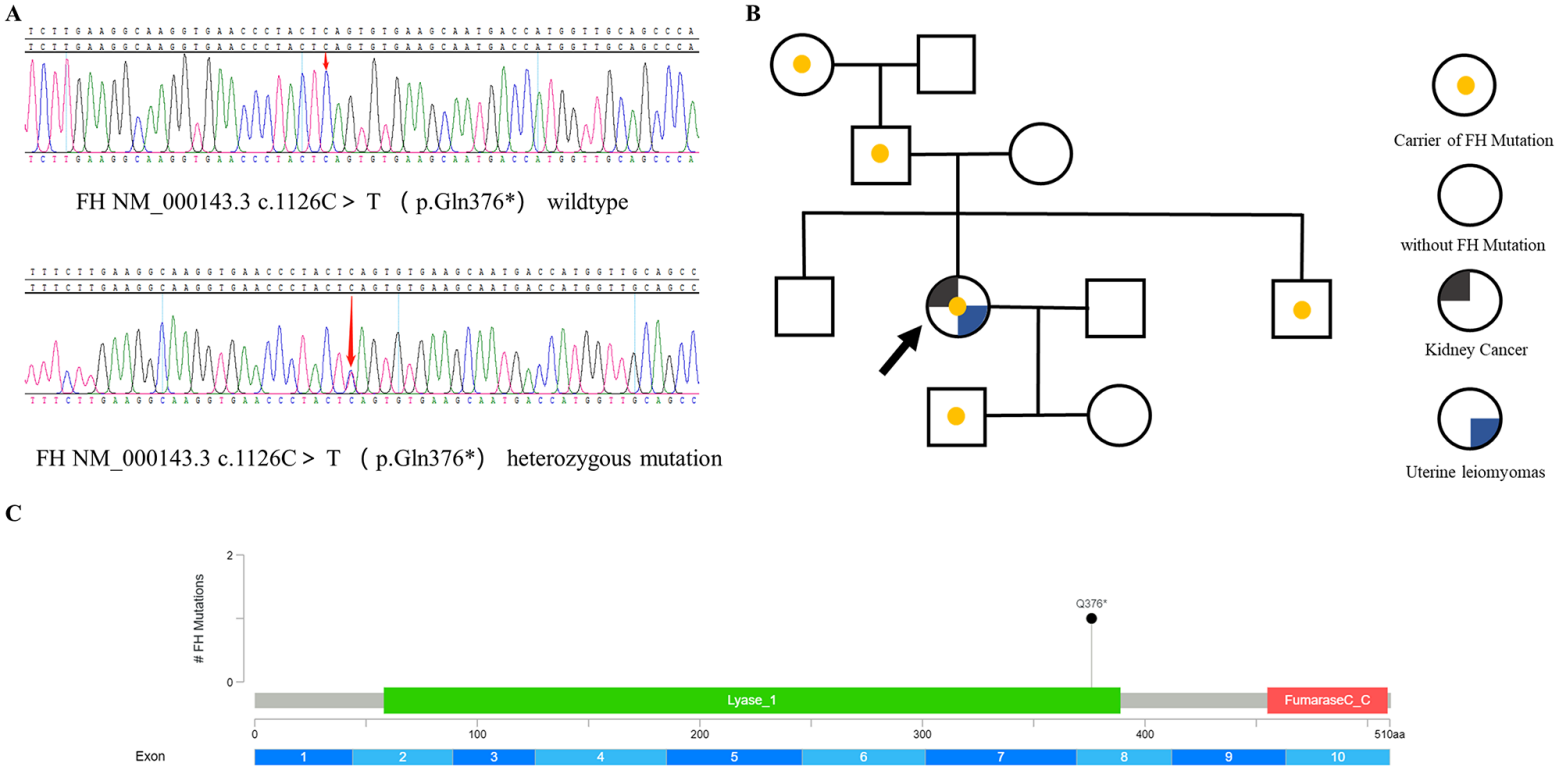
**A**. Genetic testing revealed a novel fumarate hydratase (FH) germline mutation (c.1126 C > T; p.Q376*) that was confirmed by Sanger sequencing, verifying the diagnosis of HLRCC-RCC. **B**. Pedigree of the family with the patient. The arrow indicates the proband. **C**. Graphical depiction of FH alterations in p.Q376*

A 35-year-old Chinese female presented to the hospital for cough in October 2020. A computed tomography (CT) scan revealed a large right-sided renal mass and multiple enlarged lymph nodes (Fig. 1A); the tumor was determined to be malignant. The patient subsequently underwent laparoscopic resection of the right kidney. Postoperative pathology revealed 2 type 2 papillary RCCs with papillary and tubular structures and was classified as World Health Organization/International Society of Urologic Pathologists grade III (Fig. 1B). At 3 months after surgery, CT revealed multiple nodes in both lungs suggestive of metastasis (Fig. 1C). Immunohistochemistry indicated high expression levels of PD-L1 (tumor cells + 20%, Dako 22C3) (Fig. 1D). Because she had a medical history of uterine leiomyomas in 2018, HLRCC was suspected. Next-generation sequencing of genomic DNA from tissues and blood was performed after the patient provided informed consent (Geneplus-Beijing). The sequencing results revealed a previously unidentified germline nonsense mutation in exon 8 of the FH gene (c.1126 C > T; p.Q376*). Sanger sequencing confirmed these findings (Fig. 2). Then, whole-exome sequencing of genomic DNA was performed for other members of the patient’s family, and the variant was also detected in her grandmother, father, younger brother, and son. The germline nonsense mutation was searched in the Human Gene Mutation Database (HGMD), ClinVar database, and Genome Aggregation Database. This variant was classified as a pathogenic mutation in the ClinVar database; no previous study has reported this nonsense mutation, but an FH p.Q376P germline mutation was identified and classified as pathogenic [15]. Therefore, these findings supported a diagnosis of HLRCC-RCC.

**Fig. 3:**
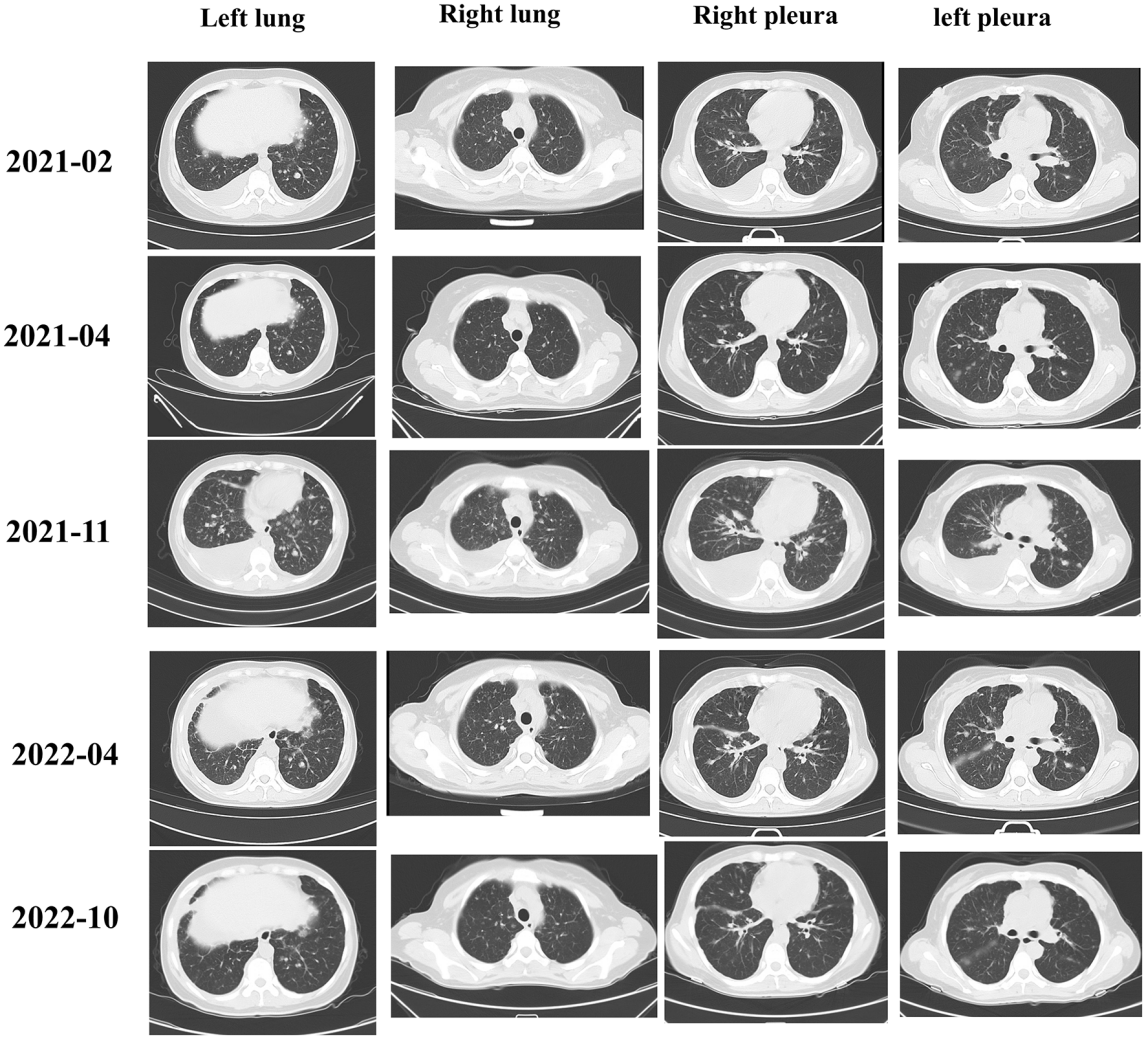
The patient had new metastatic lesions after surgery but showed a response during immunotherapy. At 3 months after surgery, CT revealed metastatic lesions in both lungs and the pleura. The lung lesions were stable until November 2021 after sintilimab and anlotinib treatment. With the switch to pembrolizumab and axitinib, the lung lesion showed a partial response in October 2022

Considering the positive expression of PD-L1, the patient was immediately treated with sintilimab and anlotinib in February 2021 (Fig. 3). Two months after treatment, a reduction in the lesion, almost to the level of a partial response, was observed. The patient’s disease was stable until November 2021. The patient subsequently experienced pleural effusion, and the other lesions were stable. The patient was switched to pembrolizumab and axitinib combined with intraperitoneal chemotherapy, and the disease was considered stable in April 2022. A partial response was observed in October 2022. The patient is still in follow-up.

We reviewed previous reports of HLRCC-RCC patients who received immunotherapy (Smethods). Five studies reported the results of immunotherapy in 76 HLRCC-RCC patients. As shown in Table 1, several case reports have confirmed the efficacy of immunotherapy or combination immunotherapy for HLRCC patients. Therefore, we further explored the correlation between different FH gene variants and immunotherapy benefits. Information on FH germline mutations and immunotherapy response in 46 HLRCC-related patients was available and collected. Of these patients, progression-free survival (PFS) of immunotherapy was available for 32 patients; and Overall survival (OS) data were available for 39 patients.

**Table 1:** The HLRCC-RCC patients with immunotherapy

Reference	Design	*N*	line	Histology (*N*)	Treatment	ORR (%)	PFS (m)	OS (m)
Wang t al [14].	Retrospective	1	1 L	HLRCC-RCC	pembrolizumab	100	NR	NR
Iribe et al. [13]	Retrospective	1	1 L	HLRCC-RCC	Nivolumab + ipilimumab	100	NR	NR
Yonese et al. [4]	Retrospective	1	> 1 L	HLRCC-RCC	Axitinib + Nivolumab	100	NR	NR
Feng et al. [16]	Retrospective	1	1 L	HLRCC-RCC	Sintilimab + Axitinib	100	NR	NR
Gleeson et al. [17]	Retrospective	3	> 1 L	HLRCC-RCC	ICI = 2 ICI + TKI = 1	ICI = 0 ICI + TKI = 100	NA	NA
Sun et al. [18]	Retrospective	5	1 L	HLRCC-RCC	ICI + TKI	16.7	13.3	NR
Lee et al. [12]	Phase 2	5	1 L	HLRCC-RCC	Cabozantinib + Nivolumab = 5	100	NA	NA
Carril et al. [11].	Retrospective	11	1 L/>1 L	HLRCC-RCC	ICI	18	2.7	NA
Xu et al. [10].	Retrospective	48	1 L/>1 L	HLRCC-RCC	ICI + TKI	33	18	NR

NA: not available.NR: not reached. N: population. ORR: overall response rate. PFS: progression-free survival. M: months. OS: overall survival. HLRCC: hereditary leiomyomatosis and renal cell cancer. HLRCC-RCC: HLRCC-associated RCC. ICI: immune checkpoint inhibitor. TKI: tyrosine kinase inhibitor

**Fig. 4:**
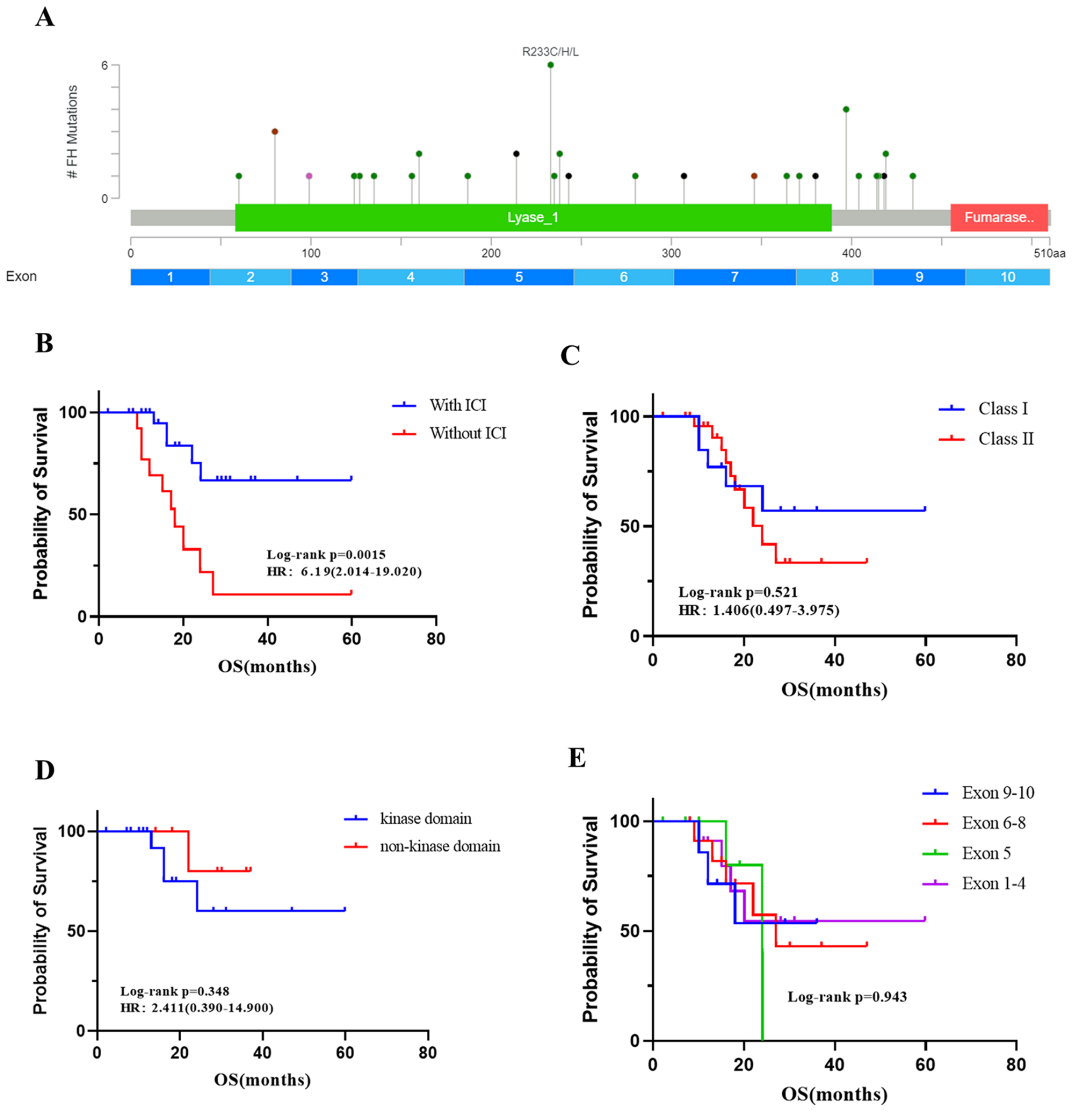
**A**. Graphical depiction of FH alterations identified in our study. **B**. The correlation between OS and ICI treatment. The correlation between OS and **C** different classes of mutations of the FH gene, **D** kinase domain mutations of the FH gene, or **E** mutations in different exons of the FH gene. ICI: immune checkpoint inhibitor

The FH germline mutations of 46 patients included 40 variants in the FH gene, including 11 insertions/deletions, 24 missense mutations, two splice site mutations, and three exon deletions (Fig. 4). In our cohort, more mutations were concentrated in exons 5, 8 and 9, accounting for 28.3%, 17.4% and 15.2%, respectively. We first analyzed the effect of immunotherapy on OS. As shown in Fig. 4, patients who received immunotherapy had a significantly better OS than those who did not (median OS: NR vs. 18.01 months, HR = 0.162, *p* = 0.0015). Then, the relationships between OS and the FH gene mutations were analyzed. No correlation was found between mutations in different exons of the FH gene and OS, as shown in Fig. 4. The OS of patients who harbored mutations in the kinase domain of the FH gene and those with mutations in the nonkinase domain were compared, and no differences were found. FH mutations with a high probability of causing protein abnormalities were defined as Class I mutations, including insertions/deletions and exon deletions; other mutations were defined as Class II mutations. However, OS did not differ between patients harboring Class I and Class II mutations. Then, we also explored whether the difference mutation of FH gene would affect the PFS of immunotherapy (SFig. 1). Patients with mutations in the FH gene kinase domain demonstrated a longer median PFS with immunotherapy compared to those with non-kinase domain mutations (18.9 vs. 16.1 months, *p* = 0.72), although this difference was not statistically significant. When comparing the PFS of patients with class I and class II FH gene mutations, patients with class I mutations also exhibited a longer median PFS, but again, the difference was not statistically significant (median: 18.9 vs. 17.0 months, *p* = 0.37). No association was found between the different exons of FH mutations and the PFS of immunotherapy. However, it was observed that patients with FH mutations in exon 5 had the longest median PFS, while those with mutations in exon 7 had the shortest PFS (18.9 months vs. 12.9 months).

## Discussion

HLRCC is a rare, autosomal dominant hereditary disorder caused by mutations in the FH gene. One manifestation of this disease is renal cell cancer, which predominantly presents as an aggressive form with a high propensity for early metastasis [19]. Treatment of these patients poses significant challenges, as there is currently no standardized therapeutic regimen; instead, treatment strategies are largely based on those employed for general renal cancer patients, often involving experimental approaches and adaptations. Here, we described a patient with HLRCC who exhibited a novel heterozygous germline FH mutation and benefited from immunotherapy. Although the patient achieved only a partial response during immunotherapy, the metastatic lesions and pleural effusion experienced long-term benefits.

HLRCC is an autosomal dominant condition caused by germline mutations in the FH gene (1q42.3–q43) [1, 20]. HLRCC patients are at risk for the development of cutaneous leiomyomas, multiple early-onset uterine leiomyomas and an aggressive form of type 2 papillary renal cell cancer [21]. Different FH mutations were detected in different HLRCC patients. To date, more than 200 variants in the FH gene, including more than 100 pathogenic mutations, have been uploaded to the Leiden Open Variant Database [22]. Moreover, the HGMD also included these reported pathogenic and suspected pathogenic FH mutations [23]. In this study, a new nonsense mutation of the FH gene was detected. Although this mutation has been documented in the ClinVar database, no relevant case has been reported to date. Considering the patient’s disease history and the mutation of the FH gene, this patient was diagnosed with HLRCC. FH gene mutations were detected by WES, and the results revealed the same mutation status across the patient’s family members. This was the first case report revealing FH p.Q376* as a pathogenic mutation.

Prompt excision of HLRCC-associated kidney tumors is critical for preventing metastasis [19]. However, no standard therapies or consensus management approaches have been established for advanced HLRCC-RCC. There have been several reports of HLRCC-RCC treatment with immunotherapy, which has attracted increasing amounts of attention as a new therapeutic option. A recent study reported the achievement of complete response in a patient with HLRCC-RCC after 31 weeks of immunotherapy combination treatment (nivolumab plus ipilimumab) [13]. In several other patients with HLRCC-RCC, immunotherapy was shown to be effective [4, 13, 14, 16]. Consistent with previous studies, our study also showed that HLRCC patients who received immunotherapy had longer OS [10]. In addition, a single-arm phase II study of cabozantinib plus nivolumab demonstrated an ORR of 100% in HLRCC patients [12]. However, several cohort studies have demonstrated that the ORR of immunotherapy ranges from 16.7 to 33% in HLRCC patients [10, 11, 17, 18]. Therefore, a total of 46 HLRCC patients who received immunotherapy were enrolled. The correlation between FH germline variation and immunotherapy response was analyzed. As in previous reports [10, 18], there was no obvious aggregation of FH mutations. Previous studies have revealed an association between FH mutations and increased genomic instability, with patients exhibiting genomic instability having a poorer prognosis [18]. Sulkowski [24] and colleagues reported that elevated levels of fumarate can inhibit the demethylation enzyme’s role in the homologous recombination DNA repair pathway, which may lead to an increase in genomic instability. However, in our analysis, there was no significant difference in treatment response among FH gene carriers across different groups, whether stratified by mutation function or mutation location. Nevertheless, due to the inability to obtain somatic mutation information of patients, we were unable to distinguish those FH germline variants that could cause genomic instability. Further research is needed to explore whether patients with FH germline mutations associated with genomic instability will benefit from immunotherapy. No impact of different types of FH germline mutations on the efficacy of immunotherapy was observed in our study. The type of FH germline mutation may not be a determining factor in whether immunotherapy is selected for the management of HLRCC-RCC patients’ diseases.

Programmed cell death-ligand 1 (PD-L1) may be a useful biomarker for immunotherapy of HLRCC. A patient who received monotherapy with pembrolizumab exhibited high expression levels of PD-L1 (30%) and achieved CR [14]. Another case reported that patients with a PD-L1 expression level of 50% achieved complete response (CR) after immunotherapy combination treatment [13]. Fortunately, our patient achieved a partial response, and the efficacy of immunotherapy and PD-L1 expression in HLRCC patients have not been investigated in cohort studies. Thus, PD-L1 may be useful as a predictor or biomarker of treatment effects in future studies. Our study has its limitations. The research data on the correlation between FH germline genes and the efficacy of immunotherapy were derived from reports of other studies, which allows us to conduct only a limited analysis. The unavailability of PD-L1 information also made it difficult to further analyze its correlation with the efficacy of immunotherapy.

## Conclusion

In conclusion, a novel FH gene mutation was identified in a patient with HLRCC-RCC. This patient benefited from immunotherapy. Moreover, our study reviewed and analyzed the efficacy of immunotherapy in HLRCC patients and suggested that immunotherapy is an effective therapeutic option for HLRCC regardless of the form of FH germline mutation.

### Electronic supplementary material

Below is the link to the electronic supplementary material.

Supplementary Material 1

Supplementary Material 2

## Data Availability

No datasets were generated or analysed during the current study.
