# Supplementary material for: Case report: response to immunotherapy and association with the fh gene in hereditary leiomyomatosis and renal cell cancer-associated renal cell cancer
Source: BMC Med Genomics. 2024 Aug 19;17:215. doi: 10.1186/s12920-024-01957-w (PMC11331603; doi:10.1186/s12920-024-01957-w)
Supplement: Supplementary file 1 — Supplementary Material 1 [file 12920_2024_1957_MOESM1_ESM.docx]

1. **Methods**

Patient was evaluated at the Henan cancer hospital. Genetic, radiological and histopathological studies were reviewed. Patient and family members gave informed consent for inclusion of their clinical data in this manuscript.

For the literature review, database of PubMed(www.ncbi.nlm.nih.gov/pubmed/, accessed on 31 August 2022) was searched for HLRCC-related renal tumors receiving immunotherapy. We searched PubMed for articles published before August 31, 2022, using “Carcinoma” OR “Cancer” OR “Tumor” AND “Renal” OR “Nephroid” OR “Kidney” OR “Papillary” OR “Hypernephroid” OR “hypernephroma” OR “RCC” AND “HLRCC” OR “Leiomyom” OR “fumarate hydratase” OR “FH mutation”. After removing duplicates, the search yielded 1669 articles. Any report (manuscript or conference abstract in English language reports) describing a HLRCC-related renal tumor and receiving immunotherapy (only consider immune checkpoint inhibitor) was eligible for inclusion. Exclusion of full text unavailability, no immunotherapy, only reviewing or describing previously reported patients, or lack of germline genetic testing to confirm the diagnosis of HLRCC, a total of 9 reports were eligible for full text screening[4, 8-15].
