# Supplementary figures and images for: Case report: response to immunotherapy and association with the fh gene in hereditary leiomyomatosis and renal cell cancer-associated renal cell cancer
Source: BMC Med Genomics. 2024 Aug 19;17:215. doi: 10.1186/s12920-024-01957-w (PMC11331603; doi:10.1186/s12920-024-01957-w)

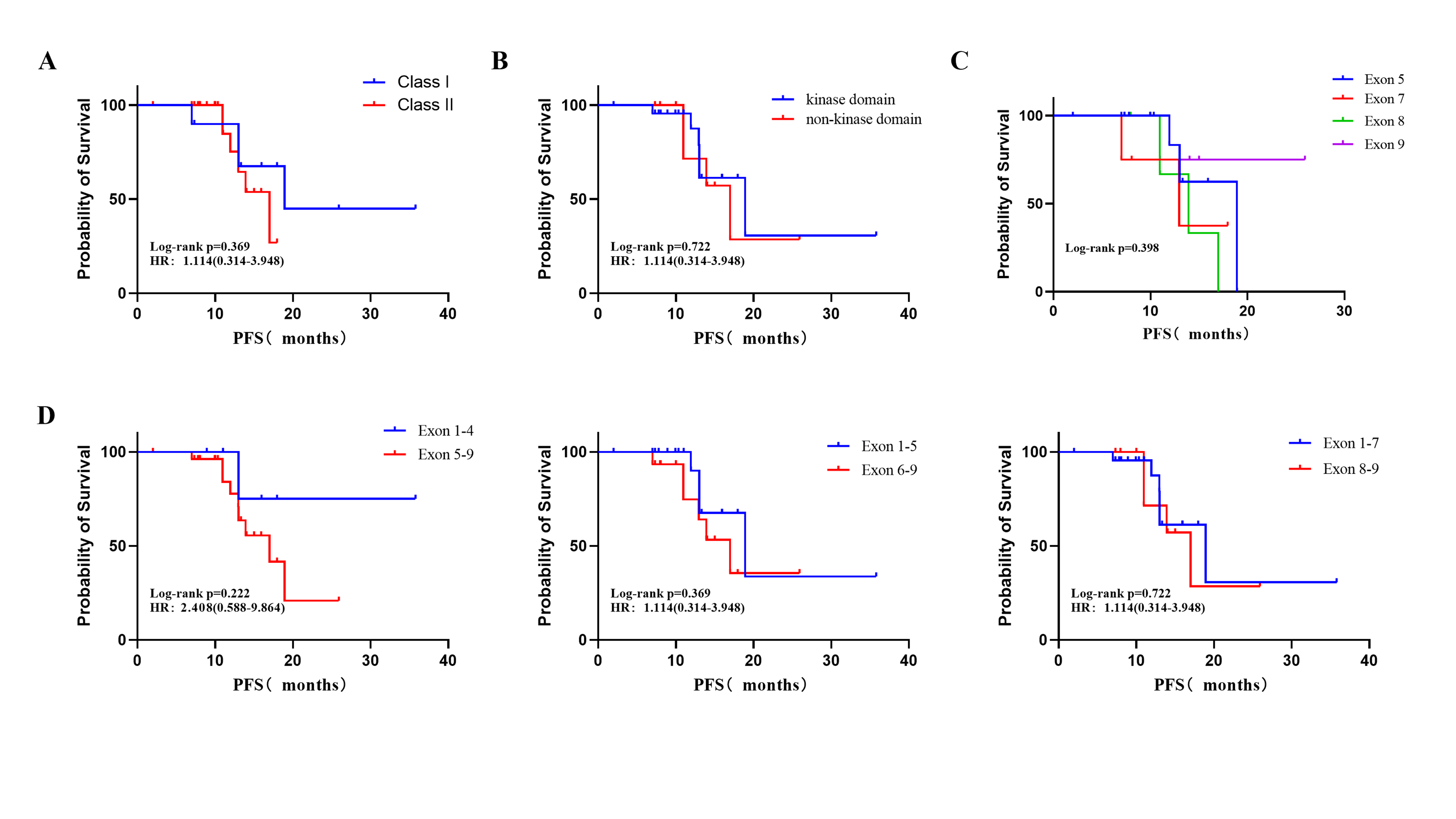

Supplement: Supplementary file 2 — Supplementary Material 2 [file 12920_2024_1957_MOESM2_ESM.tif]
